# Transcriptome Analysis Reveals Quality Improvement Mechanisms in Ratoon Rice

**DOI:** 10.3390/foods14162873

**Published:** 2025-08-19

**Authors:** Feiyan Xue, Lele Ning, Zhuangzhuang Qiang, Xiaotong Fu, Chenling Qu

**Affiliations:** School of Food and Strategic Reserves, Henan University of Technology, Zhengzhou 450001, China; 17536357849@163.com (F.X.); nll8908478123@163.com (L.N.); q13140563286@163.com (Z.Q.); 15803279802@163.com (X.F.)

**Keywords:** ratoon rice, germination, chalkiness, antioxidant enzyme activity, transcriptome

## Abstract

Ratoon rice offered higher yields and superior grain quality compared to main rice. This study compared differences in germination, chalkiness, and antioxidant enzyme activities between ratoon rice and main rice of cultivars 19X and NJXM, and analyzed the underlying quality improvement mechanisms from a transcriptome perspective. The results demonstrated that ratoon rice exhibited a significantly greater germination potential and germination rate, reduced chalky grain rate and chalkiness degree, and exhibited higher activities of superoxide dismutase (SOD) and catalase (CAT). Transcriptome analysis revealed that the enhanced germination in ratoon rice may result from significantly upregulated expression of genes qLTG3-1, OsLOX2, OsSAMDC2, and OsSAMDC4. The reduction in chalkiness in ratoon rice may involve the following three causes. First, the reduction of chalkiness may be due to the significantly upregulated GAD3 expression by enhancing high-temperature tolerance. Second, the significantly upregulated expression of peroxidase genes (prx86, POX8.1, Perox4) and significantly downregulated OsEBP89 expression potentially increased the oxidative stress tolerance to reduce the chalkiness of ratoon rice. Finally, the significantly upregulated OsNCED3 expression potentially modulated plant hormones to decrease the chalkiness of ratoon rice. These findings provided novel insights into revealing the mechanisms underlying the superior quality of ratoon rice.

## 1. Introduction

Rice (*Oryza sativa* L.) is one of the most important crops worldwide and currently serves as a staple food for more than half of the world’s population [1,2]. In these years, global population growth and industrialization have contributed to a progressive reduction in land available for rice cultivation [3]. Therefore, enhancing yields per unit area under conditions of finite arable land resources has emerged as a critical strategy to address this challenge. Ratoon rice cultivation serves as a viable agricultural intervention, with the literature demonstrating its capacity to enhance total grain production by 58.3% through optimized resource recycling from the first crop, achieving grain yields of up to 3.5 t/ha [4].

Ratooning rice refers to an agricultural practice where new panicles regenerate from remaining axillary buds on the stubble nodes following the harvest of the main crop, thereby enabling an additional harvest from the same cultivation system [5,6]. This dual-cropping mode has demonstrated significant potential for yield augmentation, minimizing requirements for both labor and agricultural land [7,8]. Additionally, the available data indicates that ratooning rice cultivation can reduce global greenhouse gas emissions and enhance the net ecosystem economic benefit of the crop [9]. It was reported that major rice-producing countries, including India, Indonesia, Iran, Japan, Nigeria, and the United States, were accelerating the promotion of ratoon rice cultivation systems [10]. As the northernmost critical zone for the distribution of this system, Japan’s Kanto region (35–36° N) was constrained by climatic conditions and the length of the growing season, resulting in the adoption of short-duration cold-tolerant japonica rice varieties. Notably, current ratoon rice practices in other parts of Asia predominantly rely on high-yielding indica or indica hybrid rice adapted to warm environments [11].

Currently, several studies have been conducted on the quality differences between ratoon rice and main rice. It was reported that the main harvest rice had a significantly higher length-to-width ratio, while its grain yield and chalkiness were significantly lower than those of ratoon rice. Additionally, the amylase content and alkali spreading value of the main rice were significantly lower than those of ratoon rice [12]. Lin et al. demonstrated that the seed setting rate, chalky grain rate, and chalkiness of ratoon rice were significantly reduced compared to the main rice. Such improvements correlated with a shift in metabolomic composition, especially regarding variations in sugar components in carbohydrate metabolism. Furthermore, rice ratooning elevated ascorbate levels and oxidative stress resilience in rice grains [2]. A previous study indicated that ratoon rice exhibited increased amylose content, decreased protein content, reduced relative crystallinity of starch, and higher hardness, cohesiveness, adhesiveness, and resilience compared to main-season rice, along with higher sensory scores [4].

Transcriptome analysis can profile all transcripts produced in plant cells and tissues, including the number of transcripts, expression dynamics at specific developmental stages, post-transcriptional modifications, and regulation of non-coding RNAs [13,14]. Transcriptome analysis has been widely applied in rice research. It primarily revealed important biological processes in rice, such as stress resistance, growth and development mechanisms, and metabolic regulation, by analyzing gene expression patterns and regulatory networks under different conditions [15]. Recent research integrating physiological, molecular, and transcriptome analyses revealed the molecular mechanisms underlying yield variation and chalkiness improvement in ratoon rice. These changes were found to be associated with the gene GF14f, which exerted negative impacts on antioxidant capacity and environmental stress resistance in ratoon rice. Furthermore, the detrimental effects of GF14f exhibited constitutive expression, being independent of seasonal or environmental conditions [16]. To elucidate the molecular mechanisms underlying antioxidant properties in rice during the post-grain filling stage, transcriptome analysis was employed to study the changes of gene expression at five distinct time points following grain filling. Through the construction of a weighted gene co-expression network (WGCNA) and modular clustering analysis of genes, specific modules exhibiting significant associations with antioxidant enzyme activities were successfully identified. Within these modules, hub genes that were significantly correlated with antioxidant traits were further characterized [17].

This study aimed to elucidate the molecular basis of quality trait differences between main rice and ratoon rice. We investigated key physiological quality parameters—including germination potential, germination rate, chalky grain incidence, and chalkiness degree—alongside antioxidative enzyme profiles (SOD, CAT activities). Utilizing transcriptome analysis, we identified differentially expressed genes (DEGs). This work could provide a theoretical foundation to support the broader promotion and application of ratoon rice systems.

## 2. Materials and Methods

### 2.1. Experimental Materials

Two indica rice cultivars, 19 Xiang and Nanjing Xiangmi, were used as experimental materials. They were cultivated in Jiangxi Province, China, with both main rice (MR) and ratoon rice (RR) harvested in August and October 2023, respectively. The two varieties of the main rice were designated as 19X-MR and NJXM-MR, and the two varieties of the ratoon rice were denoted as 19X-RR and NJXM-RR.

The intact paddy grains were selected for determining germination potential and germination rate. Subsequently, the paddy grains were dehulled using a hulling machine (Satake Corporation, Hiroshima, Japan), and brown rice samples were obtained after removing husks and broken particles. The brown rice samples can be used for antioxidant enzyme activity analysis. Some of the brown rice samples were placed in a rice milling machine (Suzhou Jiangdong Precision Instrument Co., Ltd., Suzhou, China) and the milling time was adjusted to 40 s to remove the cortex and embryo. After sieving, intact milled rice grains were collected for the determination of chalky grain rate and chalkiness degree.

### 2.2. Experimental Design

The MR and the RR were harvested in August 2023 and October 2023, respectively. After harvest, they were naturally air-dried, followed by manual removal of impurities. Then, the germination potential, germination rate, chalkiness, activities of superoxide dismutase (SOD) and catalase (CAT), as well as transcriptomics, were determined.

### 2.3. Determination of Germination Potential and Germination Rate

The determination of germination potential and germination rates was conducted with reference to the Chinese National Standard GB/T 5520-2011. Paddy grains were first soaked in water, and 100 paddy grains were then placed on each germination bed. The germination bed was cultured at 30 °C for a total of 10 days with daily watering. Germination potential referred to the percentage of normally germinated grains on the 3rd day, accounting for the number of test grains, while germination rate was the percentage of all normally germinated grains on the 10th day, accounting for the number of test grains. Four biological replicates were set for all these experiments.

### 2.4. Determination of Chalky Grain Rate and Chalkiness Degree

The chalky grain rate and chalkiness degree of rice grains were measured using a Rice Appearance Quality Tester (CanoScan 9000F MarkII, Canon, Beijing, China). Rice samples were placed on the instrument scanning platform, and then the sample images were captured.

The calculation formula for chalky grain rate was

(1) $$\mathrm{Chalky}\;\mathrm{grain}\;\mathrm{rate}=\frac{\mathrm{Number}\;\mathrm{of}\;\mathrm{chalky}\;\mathrm{grains}}{\mathrm{Total}\;\mathrm{number}\;\mathrm{of}\;\mathrm{grains}}\;\times \;100\%$$

The calculation formula for chalkiness degree was

(2) $$\mathrm{Chalkiness}\;\mathrm{degree}=\frac{\mathrm{Sum}\;\mathrm{of}\;\mathrm{chalky}\;\mathrm{area}}{\mathrm{Sum}\;\mathrm{of}\;\mathrm{whole}\;\mathrm{grain}\;\mathrm{area}}\;\times \;100\%$$

### 2.5. Determination of SOD Activity

The activity of superoxide dismutase (SOD) was measured using a commercial kit (Solarbio Life Science, BC 0170, Beijing, China). A total of 0.1 g of brown rice flour was homogenized with 1 mL of phosphate-buffered saline (PBS) on ice. The homogenate was centrifuged at 8000× *g* for 10 min at 4 °C. The resulting supernatant was collected and maintained on ice for subsequent analysis. For superoxide dismutase (SOD) activity determination, the supernatant was incubated with nitroblue tetrazolium (from the assay kit) at 37 °C for 30 min. The absorbance was immediately measured at 560 nm using a spectrophotometer (T700A UV-Vis Spectrophotometer, Beijing Purkinje General Instrument Co., Ltd., Beijing, China). SOD activity was calculated according to the manufacturer’s protocol based on the recorded absorbance values.

### 2.6. Determination of CAT Activity

The activity of catalase (CAT) was measured using a commercial kit (Solarbio Life Science, BC 0200, Beijing, China). Specifically, 0.1 g of brown rice flour was weighed and homogenized with 1 mL of extraction buffer in an ice bath. The homogenate was centrifuged at 8000× *g* for 10 min at 4 °C, and the supernatant was collected and stored on ice for subsequent analysis. For catalase (CAT) activity determination, a CAT assay working solution was prepared according to the manufacturer’s specifications (volume-adjusted as needed). The working solution was equilibrated at 25 °C for 10 min. Subsequently, 1 mL of the working solution was transferred to a 1 mL quartz cuvette, followed by the addition of a 35 μL sample. The mixture was immediately mixed by vortexing for 5 s. Absorbance measurements were recorded at 240 nm immediately (t = 0) and after 1 min (t = 1 min) using a spectrophotometer (T700A UV-Vis Spectrophotometer, Beijing Purkinje General Instrument Co., Ltd., Beijing, China) at ambient temperature. CAT activity was calculated from the absorbance difference according to the kit manufacturer’s protocol.

### 2.7. Determination of Transcriptomics

Total RNA was isolated from fresh rice (0.1 g per sample, immediately frozen in liquid nitrogen after sampling and stored at −80 °C) using the TRIeasy Plus RNA Kit (YeaSen, Shanghai, China) [18]. To ensure RNA quality, RNA integrity was assessed by 1.2% agarose gel electrophoresis (120 V for 30 min), purity was determined using a NanoPhotometer spectrophotometer (Implen, Munich, Germany) with an acceptable OD260/280 ratio of 1.8–2.0, concentration was measured via a Qubit 4.0 fluorometer (Invitrogen, Carlsbad, CA, USA), and the RNA integrity number (RIN) was evaluated using a Qsep400 bioanalyzer (BiOptic, New Taipei City, Taiwan), with a passing threshold of RIN ≥ 7.0. mRNA with polyA tails was enriched using Oligo (dT) magnetic beads (Thermo Fisher, Waltham, MA, USA). Subsequently, RNA was fragmented into short fragments by adding fragmentation buffer (Illumina, San Diego, CA, USA, catalog number RS-122-2103).

Using the short fragment RNA as the template, first-strand cDNA was synthesized with hexamer random primers (Thermo Fisher, Waltham, MA, USA). Then, second-strand cDNA was generated by adding buffer, dNTPs (including dTTP, dATP, dGTP, and dCTP; Takara, Beijing, China), and DNA polymerase (New England Biolabs, Ipswich, MA, USA) I. Double-stranded cDNA was purified using DNA purification magnetic beads (Beckman, Brea, CA, USA). The purified double-stranded cDNA was subjected to end repair, adenylation, and ligation of sequencing adaptors (Illumina). Size selection was performed using DNA purification magnetic beads. Finally, the final cDNA library was obtained via PCR amplification. After passing quality inspection (concentration ≥ 10 ng/μL and expected fragment distribution), sequencing was performed on the Illumina NovaSeq 6000 platform, with three independent biological replicates set for each treatment.

### 2.8. Statistical Analysis

Four biological replicates were set for the determination of germination potential and germination rate, while three replicates were used for the detection of chalky grain rate, chalkiness degree, superoxide dismutase (SOD) and catalase (CAT) activities, as well as transcriptome analysis.

Statistical analyses were performed using SPSS 27.0 software (IBM Corp., USA) through analysis of variance (ANOVA) followed by Duncan’s multiple range test, with differences considered statistically significant at the 95% confidence level (*p* < 0.05). Germination potential and germination rate, chalky grain rate and chalkiness degree, superoxide dismutase (SOD), and catalase (CAT) activity graphs were generated using Origin 2021, while transcriptome analysis graphs were created using the online platform of Weishengxin (https://www.bioinformatics.com.cn/, accessed on 10 May 2025).

Gene function analysis was carried out through the National Center for Biotechnology Information (NCBI, https://www.ncbi.nlm.nih.gov/, accessed on 20 May 2025), the Rice Annotation Project Database (https://rapdb.dna.affrc.go.jp/index.html, accessed on 25 May 2025), and the National Rice Database of the National Rice Data Center (https://www.ricedata.cn/reference/, accessed on 1 June 2025); the data access dates were from 10 May 2025 to 10 June 2025.

## 3. Results

### 3.1. Comparison of Germination Potential (GP) and Germination Rate (GR) Between Main Rice and Ratoon Rice

**Figure 1:**
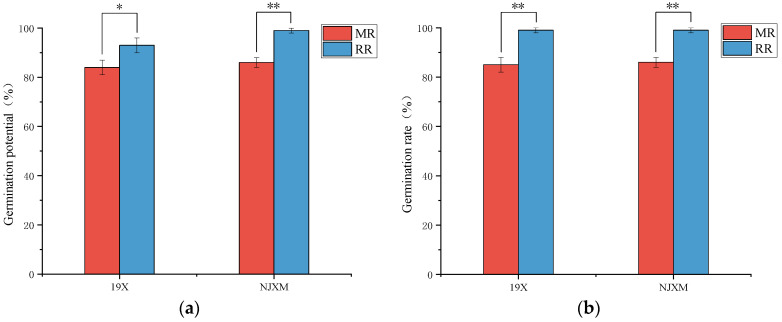
Comparison of (**a**) germination potential and (**b**) germination rate between main rice (MR, red bars) and ratoon rice (RR, blue bars) for two varieties: 19X and NJXM. “*” indicated *p* < 0.05, “**” indicated *p* < 0.01.

Seed GP and GR were key indicators for assessing seed quality and germination capacity while serving as a critical metric for evaluating growth potential and plant growing performance [19]. As illustrated in Figure 1, both 19X-RR and NJXM-RR demonstrated significantly higher GP and GR compared to their main rice (*p* < 0.05) counterparts. Collectively, these results confirmed ratoon rice’s superior seed germination traits.

### 3.2. Comparison of Chalky Grain Rate and Chalkiness Degree Between Main Rice and Ratoon Rice

**Figure 2:**
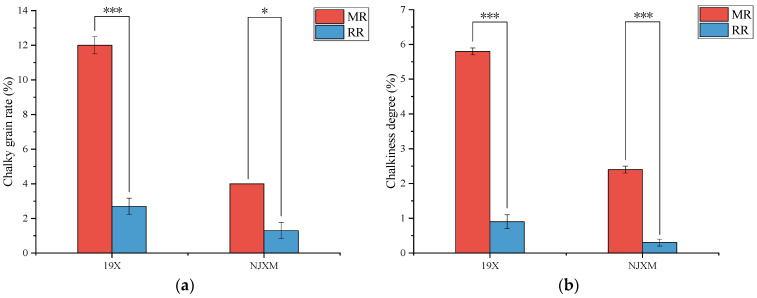
Comparison of (**a**) chalky grain rate and (**b**) chalkiness degree between main rice (MR, red bars) and ratoon rice (RR, blue bars) for two varieties: 19X and NJXM. “*” indicated *p* < 0.05, “***” indicated *p* < 0.001.

Chalkiness referred to the opaque regions in rice grains. Additionally, chalkiness increased the brittleness of rice grains and reduced their market value [20]. As shown in Figure 2, both 19X and NJXM ratoon rice exhibited significantly lower chalky grain rate and chalkiness degree than the main rice (*p* < 0.05). These results demonstrated significantly superior grain appearance quality in ratoon rice. Alizadeh [12] and Huang [21] et al. had also reported that the chalkiness of ratoon rice was significantly lower than that of main rice, which was consistent with the findings of our study.

### 3.3. Comparison of Superoxide Dismutase (SOD) and Catalase (CAT) Activities Between Main Rice and Ratoon Rice

**Figure 3:**
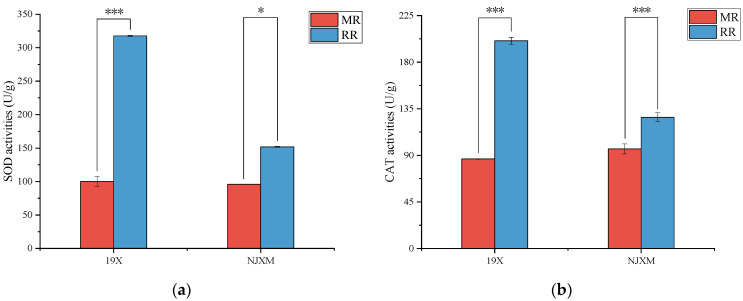
Comparison of (**a**) SOD activity and (**b**) CAT activity between main rice (MR, red bars) and ratoon rice (RR, blue bars) for two varieties: 19X and NJXM. “*” indicated *p* < 0.05, “***” indicated *p* < 0.001.

Superoxide dismutase (SOD) and catalase (CAT) are key antioxidant enzymes in plants, playing essential roles in their metabolic activities. Their primary functions include scavenging reactive oxygen species (ROS), maintaining redox balance, and protecting cells from oxidative damage [22]. SOD serves as the first line of defense in the plant antioxidant system, functioning to catalyze the conversion of superoxide anion radicals (O_2_^−^) into hydrogen peroxide (H_2_O_2_) and oxygen (O_2_), thereby reducing the accumulation of reactive oxygen species (ROS) [23]. CAT is responsible for decomposing hydrogen peroxide (H_2_O_2_) into water and oxygen, thereby preventing the excessive accumulation of H_2_O_2_ in cells [24]. As shown in Figure 3, both 19X-RR and NJXM-RR exhibited significantly higher superoxide dismutase (SOD) and catalase (CAT) activities than the main rice (*p* < 0.05).

### 3.4. Transcriptome Analysis

To investigate the differences in quality traits between main rice and ratoon rice, this study employed a transcriptomic approach using high-throughput RNA sequencing to identify differentially expressed genes (DEGs) in the main and ratoon rice of the two paddy varieties.

#### 3.4.1. Quality Analysis of Transcriptome Sequencing

In this experiment, transcriptome sequencing analysis was performed on 12 test samples, including four groups of samples (ratoon rice and main rice of two varieties) with three biological replicates each. A total of 648,859,190 raw reads (raw data read counts) were obtained, and 614,027,496 clean reads (high-quality read counts after raw data filtering) were generated after filtering. The average values of Q20 (the percentage of bases with a Qphred score ≥ 20 in the total number of bases) and Q30 (the percentage of bases with a Qphred score ≥ 30 in the total number of bases) for the 12 samples were 97.75% and 94.13%, respectively, and the mean GC content (the percentage of the sum of guanine G and cytosine C counts in high-quality reads to the total number of bases) was 54.13% (Table 1). This indicated that the quality of transcriptome sequencing data met the standards for differentially expressed gene analysis.

#### 3.4.2. Analysis of Differentially Expressed Genes (DEGs)

**Figure 4:**
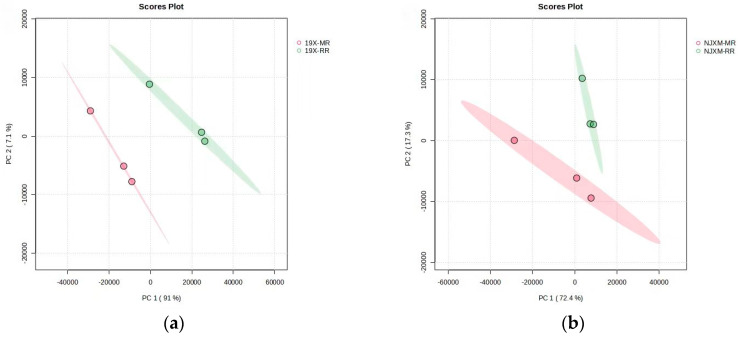
Comparison of differential gene expression between main rice and ratoon rice. (**a**) PCA plot of 19X main rice and ratoon rice; (**b**) PCA plot of NJXM main rice and ratoon rice. PC1 and PC2 represent the first and second principal components, respectively. Each scatter point stands for an individual sample. The plots revealed substantial differences in gene expression between the main rice and ratoon rice of both 19X (left plot) and NJXM (right plot) varieties, as samples of different rice types (distinguished by colors) showed clear separation along the PC1–PC2 axes.

Principal Component Analysis (PCA), a statistical method for analyzing multi-dimensional data in unsupervised pattern recognition, was employed to assess the overall differences in gene expression between main rice and ratoon rice. The PCA results for the two groups of samples are shown in Figure 4a,b, where PC1 and PC2 denote the first and second principal components, respectively. Each scatter point represents an individual sample, revealing substantial differences in gene expression between the main rice and ratoon rice of both 19X and NJXM varieties. To screen for genes with significantly differential expression, thresholds of fold change (FC) ≥ 2.0 or ≤0.5 and false discovery rate (FDR) < 0.05 were applied [25] (FDR refers to the false discovery rate after multiple hypothesis testing correction for *p*-values.).

**Figure 5:**
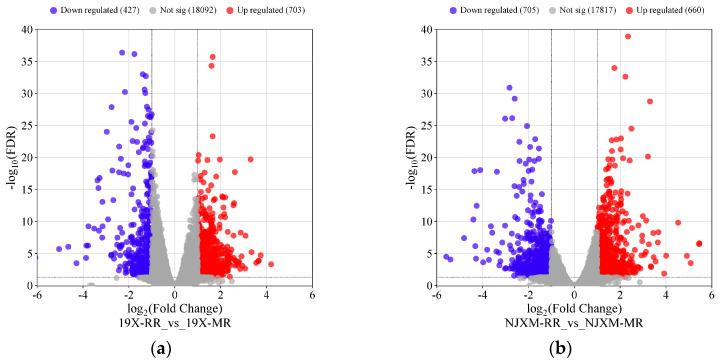
Comparison of differential gene expression between main rice and ratoon rice (**a**) Volcano plot of 19X main rice and ratoon rice (**b**) Volcano plot of NJXM main rice and ratoon rice. Red points denote genes with significantly upregulated expression levels, blue points indicate genes with significantly downregulated expression levels, and gray points represent genes with non-significant expression.

**Figure 6:**
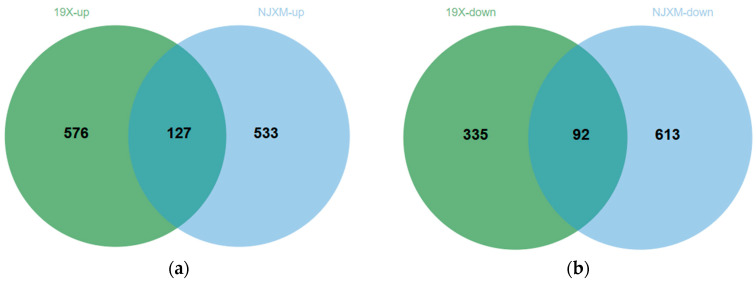
Intersection of differentially expressed genes (DEGs) in ratoon rice between varieties 19X and NJXM. (**a**) Upregulated DEGs. The Venn diagram shows 576 upregulated gene expressions for 19X, 533 for NJXM, and 127 shared by both varieties. (**b**) Downregulated DEGs. The Venn diagram shows 335 downregulated gene expressions for 19X, 613 for NJXM, and 92 shared by both varieties.

The results of the screening are presented in Figure 5a,b, where each point represents a gene. In the comparison of the main and ratoon rice of 19X, a total of 703 genes showed significantly upregulated expression levels, while 427 genes showed significantly downregulated expression. For NJXM, the comparison between main and ratoon rice identified 660 significantly upregulated expression genes and 705 significantly downregulated expression genes. Subsequently, we compared the DEGs from 19X and NJXM to find genes consistently differentially expressed in both varieties. The results are presented in Figure 6a,b. Similarly, 127 common upregulated DEGs and 92 common downregulated DEGs were identified, which showed a total of 219 common DEGs for 19X-RR vs. 19X-MR and NJXM-RR vs. NJXM-MR.

#### 3.4.3. Effects of Related Genes on Germination of Main Rice and Ratoon Rice

As indicated in Results 3.1, both the germination potential and germination rate of ratoon rice were significantly improved compared with those of main rice. As presented in Section 3.4.2, comparative transcriptome analysis identified 219 DEGs in both 19X and NJXM. Through systematic integration of the NCBI database, Rice Annotation Project Database (RAP-DB), National Rice Data Center, and the literature, four seed germination-associated genes were screened out. All these four genes demonstrated upregulated expression.

As shown in RAP-DB, the gene *qLTG3-1* (Os03g0103300) was related to low-temperature germinability and pre-harvest sprouting resistance. It functions as a major regulator of germination potential in rice, participating in seed germination processes [26]. The expression of gene *qLTG3-1* exhibited significant upregulation in both cultivars, with 2.24-fold and 2.04-fold higher expression in 19X-RR vs. 19X-MR and NJXM-RR vs. NJXM-MR, respectively. As shown in RAP-DB, the expression of gene *OsLOX2* (Os03g0738600) was also critically involved in seed germination and longevity. Huang et al. [27] employed overexpression and knockout approaches to elucidate the role of the rice *OsLOX2* gene in seed germination. Their results demonstrated that enhanced *OsLOX2* expression in transgenic lines led to significantly higher germination rates. Conversely, reduced *OsLOX2* expression resulted in slower germination. As shown in Table 2, the expression of gene *OsLOX2* showed significant upregulation in both 19X-RR vs. 19X-MR and NJXM-RR vs. NJXM-MR, with fold change values of 5.92 and 9.26, respectively. Therefore, upregulation of this gene expression would accelerate germination and enhance the germination rate of ratoon rice seeds.

Genes *OsSAMDC2* (OS02G0611200) and *OsSAMDC4* (OS09G0424300) were also implicated in seed germination. As indicated in RAP-DB, the genes *OsSAMDC2* (Os02g0611200) and *OsSAMDC4* (Os09g0424300) both encoded S-adenosylmethionine decarboxylase; additionally, *OsSAMDC2* was involved in polyamine biosynthesis and played a role in plant resistance to abiotic stresses. Chen [28] et al. generated transgenic rice plants with *OsSAMDC2* and *OsSAMDC4* knockdown via an antisense RNA interference (RNAi) strategy. Their study revealed that the transgenic lines exhibited reduced plant height, significantly decreased seed germination rates and final germination percentages, diminished pollen viability, and reduced tolerance to abiotic stresses. As shown in Table 2, the expression of genes *OsSAMDC2* and *OsSAMDC4* both exhibited significant upregulation in both cultivars, with a 2.68-fold and 3.87-fold higher expression in 19X-RR vs. 19X-MR, and 2.57-fold and 3.33-fold higher expression in NJXM-RR vs. NJXM-MR, respectively. Therefore, upregulation of *OsSAMDC2* and *OsSAMDC4* in ratoon rice may enhance the germination rate of its seeds.

#### 3.4.4. Effects of Related Genes on Chalkiness of Main Rice and Ratoon Rice

As indicated in Results 3.2, compared with the main rice varieties, the chalky grain rate and the chalkiness degree of both 19X-RR and NJXM-RR were significantly reduced. Section 3.4.2 reported 219 DEGs of 19X and NJXM cultivars. An integrated screening framework incorporating the NCBI database, Rice Annotation Project Database (RAP-DB), National Rice Data Center, and the literature identified six seed chalkiness-associated genes, with five genes exhibiting upregulated expression and one showing downregulated expression. Chalkiness degree was a complex trait controlled by multiple genes and was highly susceptible to environmental influences [29].

Research has shown that high temperature/heat stress was a critical factor inducing chalkiness in rice grains by affecting the starch content and composition, changing the fine structure of amylopectin, and increasing the ratio of long chains to short chains of amylopectin [30]. As revealed by RAP-DB, the gene *GAD3* (Os03g0236200) encoded glutamate decarboxylase. As reported in the relevant literature, its expression was regulated by the transcription factor *OsMYB55*, closely related to rice thermotolerance [31]. As shown in Table 2, the expression of gene *GAD3* showed significant upregulation in both 19X-RR vs. 19X-MR and NJXM-RR vs. NJXM-MR, with fold change values of 2.21 and 2.38, respectively. Therefore, the upregulation expression of this gene in ratoon rice may enhance its tolerance to high-temperature stress, thereby reducing chalkiness.

Moreover, reactive oxygen species ROS produced under heat stress were involved in α-amylase induction in maturating rice grains through the regulating of the levels of gibberellin (GA) and abscisic acids (ABA), ultimately contributing to grain chalkiness [32]. ROS can also be produced under other stresses, such as wounding, pathogen attack, and oxidative stress [33]. In RAP-DB, gene *Prx86* (Os06g0547400) was described as similar to Peroxidase P7, while gene *POX8.1* (Os07g0677100) was described as a peroxidase, and gene *Perox4* (Os07g0677200) was described as a peroxidase involved in the negative regulation of rice resistance to blast disease. These genes play a role in responding to environmental stresses by participating in the process of hydrogen peroxide (H_2_O_2_) scavenging [34]. As shown in Table 2, the expressions of genes *Prx86*, *POX8.1*, and *Perox4* all exhibited significant upregulation in both cultivars, with 2.06-fold, 5.93-fold, and 9.88-fold higher expression in 19X-RR vs. 19X-MR, and 2.55-fold, 5.69-fold, and 3.59-fold higher expression in NJXM-RR vs. NJXM-MR, respectively. The upregulation expression of these genes in ratoon rice suggested that ratooning may enhance its tolerance to oxidative stress.

In RAP-DB, the gene *OsEBP89* (Os03g0182800) was described as an APETALA2/ethylene responsive factor belonging to the ERF transcription factor family, which was associated with tolerance to drought and submergence stress. Studies have demonstrated that *OsEBP89* (Os03g0182800) knockout lines exhibit enhanced reactive oxygen species (ROS) scavenging capacity [35]. As shown in Table 2, the expression of gene *OsEBP89* showed significant downregulation in both 19X-RR vs. 19X-MR and NJXM-RR vs. NJXM-MR, with fold change values of 0.24 and 0.27, respectively. Consequently, the downregulation of *OsEBP89* in ratoon rice may reduce its chalkiness by improving tolerance to oxidative stress. The activities of SOD and CAT were significantly higher for ratoon rice (showed in Section 3.3). This indicates that the increased antioxidant enzyme activity may mitigate oxidative damage to the endosperm structure, thereby reducing chalkiness.

Furthermore, it was reported that phytohormones, particularly ABA and auxin, were involved in the regulation of rice chalkiness formation through the interaction of multiple transcription factors and their downstream regulatory factors [36]. As indicated in RAP-DB, *OsNCED3* (Os03g0645900), encoded 9-cis-epoxycarotenoid dioxygenase serves as a key enzyme in ABA biosynthesis. Overexpression of *OsNCED3* enhanced ABA content, reduced gibberellin (GA) levels, elevated the ABA/GA ratio, and improved tolerance to water stress [37], whereas knockout of *OsNCED3* led to the opposite outcomes. As shown in Table 2, the expression of gene *OsNCED3* exhibited significant upregulation in both cultivars, with 3.91-fold and 3.96-fold higher expression in 19X-RR vs. 19X-MR and NJXM-RR vs. NJXM-MR, respectively. In ratoon rice, upregulation expression of the *OsNCED3* gene can also lead to chalkiness reduction.

**Table 2:** Summary of gene expression *p*-values and fold change (FC) values.

Indicator	Gene Name	^1^ *p*-Value (19X)	^1^ *p*-Value (NJXM)	^2^ Fold Change (19X)	^2^ Fold Change (NJXM)
Germination	qLTG3-1 (Os03g0103300)	0.000000527	0.000000841	2.24	2.04
	OsLOX2 (Os03g0738600)	0.000000000	0.000000000	5.92	9.26
	OsSAMDC2 (OS02g0611200)	0.000000000	0.000010445	2.68	2.57
	OsSAMDC4 (OS09g0424300)	0.000000000	0.000035246	3.87	3.33
Chalkiness	GAD3 (Os03g0236200)	0.000000003	0.000000175	2.21	2.38
	prx86 (Os06g0547400)	0.000000061	0.000000000	2.06	2.55
	POX8.1 (OS07G0677100)	0.000002788	0.000000667	5.93	5.69
	Perox4 (OS07G0677200)	0.000000000	0.000000046	9.88	3.59
	OsEBP89 (Os03g0182800)	0.000000000	0.000000001	0.24	0.27
	OsNCED3 (Os03g0645900)	0.000000000	0.000000001	3.91	3.96

^1^ *p*-value: Statistical significance of differential expression (adjusted for multiple testing). ^2^ Fold change (FC): Magnitude and direction of change (FC > 1 = upregulated; FC < 1 = downregulated).

## 4. Conclusions

The results indicated that the ratoon rice of cultivars 19X and NJXM exhibited significant improvement in physiological activity and appearance quality over their main rice varieties. The ratoon rice displayed enhanced germination ability, reduced chalkiness, and increased antioxidant enzyme activity. Transcriptome analysis identified key gene candidates and global expression trends correlating with quality improvements in ratoon rice. Enhanced germination was associated with the upregulated expression of genes *qLTG3-1*, *OsLOX2*, *OsSAMDC2*, and *OsSAMDC4*. The reduction in chalkiness involved the coordinated effects of the upregulated expression of genes *GAD3*, *prx86*, *POX8.1*, *Perox4*, and *OsNCED3*, coupled with the downregulated expression of *OsEBP89*. The findings of this study established a theoretical foundation for the improvement of ratoon rice quality and the advancement of high-efficiency cultivation practices.

## Figures and Tables

**Table 1:** Sequencing data quality statistics.

^1^ Sample	^2^ Raw Reads	^3^ Clean Reads	^4^ Clean Base (G)	^5^ Q20 (%)	^6^ Q30 (%)	^7^ GC Content %
19X1-1	56,681,894	53,736,944	8.06	97.69	94.04	54.95
19X1-2	54,061,666	50,933,190	7.64	97.79	94.27	54.60
19X1-3	50,917,028	48,219,568	7.23	97.71	94.04	54.21
19X2-1	53,420,054	50,697,788	7.60	97.82	94.27	54.11
19X2-2	51,073,464	48,442,364	7.27	97.66	94.94	54.86
19X2-3	53,402,892	50,456,662	7.57	97.84	94.20	53.51
NJXM1-1	58,398,026	55,019,038	8.25	97.71	94.07	54.51
NJXM1-2	52,317,108	49,315,350	7.40	97.81	94.17	54.75
NJXM1-3	55,880,982	52,427,952	7.86	97.70	94.07	54.53
NJXM2-1	55,335,660	52,339,804	7.85	97.74	94.13	55.56
NJXM2-2	56,037,472	53,674,624	8.05	97.86	94.28	53.29
NJXM2-3	51,332,944	48,764,212	7.31	97.71	94.05	55.32

^1^ Sample: 19X1 denoted the main rice of 19X, 19X2 denoted the ratoon rice of 19X; NJXM1 denoted the main rice of NJXM, NJXM2 denoted the ratoon rice of NJXM. ^2^ Raw Reads: Raw data read counts. ^3^ Clean Reads: High-quality read counts after raw data filtering. ^4^ Clean Bases: Total base count of high-quality reads. ^5^ Q20: The percentage of bases with a Qphred score ≥ 20 in the total number of bases. ^6^ Q30: The percentage of bases with a Qphred score ≥ 30 in the total number of bases. ^7^ GC Content: The percentage of the sum of guanine G and cytosine C counts in high-quality reads to the total number of bases.

## Data Availability

The original contributions presented in the study are included in the article, further inquiries can be directed to the corresponding author.
